# Supplementary material for: A Likelihood Approach for Real-Time Calibration of Stochastic Compartmental Epidemic Models
Source: PLoS Comput Biol. 2017 Jan 17;13(1):e1005257. doi: 10.1371/journal.pcbi.1005257 (PMC5240920; doi:10.1371/journal.pcbi.1005257)
Supplement: S1 File — (TAR.GZ) [file pcbi.1005257.s014.tar.gz › HSPH_Online-SI-Revision/MSS10/n100-extreme/n100-extreme_NEW2_table4.pdf]

1	newly 3 weeks spec	2392.	1943.62	{1889., 2003.}	18.745	0.0902596	newly 3 weeks cumul	9902.	8546.26	{8465., 8622.}	13.6916	0.063957	newly 5 weeks spec	1155.	946.	{893., 994.}	18.0952	0.0870295	newly 5 weeks cumul	12 790.	10 849.	{10 758., 10 937.}	15.1761	0.0714904	newly 50 weeks cumul	15 830.	12 978.2	{12 856., 13 080.}	18.0153	0.086278
2	newly 3 weeks spec	2435.	2322.32	{2258., 2378.}	4.62752	0.0206596	newly 3 weeks cumul	9225.	8978.34	{8883., 9055.}	2.67382	0.0117825	newly 5 weeks spec	1337.	1409.22	{1356., 1463.}	5.47345	0.0229671	newly 5 weeks cumul	12 421.	12 199.5	{12 073., 12 325.}	1.78367	0.00782768	newly 46 weeks cumul	16 331.	16 790.4	{16 616., 16 961.}	2.81293	0.0120337
3	newly 3 weeks spec	1444.	1427.3	{1381., 1477.}	2.29086	0.0100628	newly 3 weeks cumul	6755.	6614.	{6541., 6685.}	2.0983	0.00922595	newly 5 weeks spec	609.	646.7	{609., 684.}	6.56486	0.0273014	newly 5 weeks cumul	8307.	8227.4	{8129., 8308.}	1.04033	0.00455188	newly 51 weeks cumul	9551.	9540.31	{9427., 9643.}	0.70223	0.00305312
4	newly 3 weeks spec	766.	790.66	{755., 823.}	4.08616	0.017033	newly 3 weeks cumul	4576.	4474.98	{4382., 4512.}	3.27622	0.0142383	newly 5 weeks spec	254.	260.52	{235., 287.}	7.0315	0.0296821	newly 5 weeks cumul	5299.	5187.84	{5086., 5224.}	3.22061	0.0139641	newly 55 weeks cumul	5634.	5542.01	{5425., 5596.}	2.82162	0.0121679
5	newly 3 weeks spec	1978.	1950.13	{1890., 2003.}	2.01466	0.00886834	newly 3 weeks cumul	9313.	8990.32	{8890., 9062.}	3.46483	0.0153265	newly 5 weeks spec	894.	878.16	{840., 914.}	2.98434	0.0132127	newly 5 weeks cumul	11 542.	11 180.7	{11 095., 11 267.}	3.13022	0.0138203	newly 52 weeks cumul	13 327.	12 935.1	{12 795., 13 055.}	2.94057	0.012974
6	newly 3 weeks spec	2370.	2328.82	{2264., 2393.}	2.48523	0.0109223	newly 3 weeks cumul	9890.	9986.92	{9882., 10 067.}	1.07219	0.00459681	newly 5 weeks spec	1132.	1184.1	{1128., 1229.}	4.96996	0.0208798	newly 5 weeks cumul	12 727.	12 831.1	{12 710., 12 901.}	0.902019	0.00386537	newly 50 weeks cumul	15 600.	15 674.2	{15 538., 15 760.}	0.653077	0.0027886
7	newly 3 weeks spec	1951.	1787.11	{1722., 1844.}	8.40031	0.0382385	newly 3 weeks cumul	8547.	7620.43	{7540., 7699.}	10.8409	0.0498508	newly 5 weeks spec	968.	929.6	{891., 973.}	4.28099	0.0191858	newly 5 weeks cumul	10 776.	9840.28	{9762., 9920.}	8.68337	0.0394612	newly 48 weeks cumul	12 929.	12 176.9	{12 056., 12 289.}	5.81731	0.0260412
8	newly 3 weeks spec	2287.	2410.27	{2350., 2474.}	5.39003	0.0227246	newly 3 weeks cumul	9859.	10 054.3	{9978., 10 137.}	1.98113	0.00851064	newly 5 weeks spec	1178.	1285.25	{1235., 1343.}	9.10441	0.0376268	newly 5 weeks cumul	12 762.	13 097.7	{13 021., 13 185.}	2.6307	0.0112711	newly 50 weeks cumul	15 717.	16 466.7	{16 364., 16 574.}	4.76993	0.0202321
9	newly 3 weeks spec	2292.	1658.14	{1608., 1708.}	27.6553	0.140713	newly 3 weeks cumul	10 287.	7890.47	{7807., 7981.}	23.2967	0.115202	newly 5 weeks spec	1160.	717.22	{676., 753.}	38.1707	0.209154	newly 5 weeks cumul	13 098.	9700.53	{9612., 9797.}	25.9388	0.130423	newly 50 weeks cumul	15 554.	11 043.8	{10 905., 11 160.}	28.9971	0.148741
10	newly 3 weeks spec	296.	293.8	{260., 332.}	7.04054	0.0308892	newly 3 weeks cumul	1966.	1942.66	{1841., 2044.}	3.6999	0.0162757	newly 5 weeks spec	92.	78.18	{61., 97.}	18.5435	0.0930038	newly 5 weeks cumul	2237.	2172.14	{2053., 2300.}	4.33795	0.0193387	newly 54 weeks cumul	2337.	2255.37	{2128., 2376.}	4.80488	0.0215018
11	newly 3 weeks spec	2110.	2393.79	{2336., 2453.}	13.4498	0.0547061	newly 3 weeks cumul	9055.	9827.46	{9714., 9939.}	8.53076	0.0355373	newly 5 weeks spec	1137.	1316.62	{1271., 1371.}	15.7977	0.0634963	newly 5 weeks cumul	11 827.	12 925.	{12 766., 13 055.}	9.28359	0.0385422	newly 48 weeks cumul	14 734.	16 440.7	{16 304., 16 586.}	11.5835	0.0475901
12	newly 3 weeks spec	2018.	1351.32	{1303., 1398.}	33.0367	0.174348	newly 3 weeks cumul	9115.	6305.13	{6223., 6376.}	30.8269	0.160081	newly 5 weeks spec	959.	613.42	{573., 651.}	36.0355	0.194523	newly 5 weeks cumul	11 517.	7837.01	{7734., 7932.}	31.9527	0.167207	newly 49 weeks cumul	13 641.	9053.51	{8942., 9167.}	33.6302	0.178049
13	newly 3 weeks spec	3118.	1899.28	{1834., 1958.}	39.0866	0.215429	newly 3 weeks cumul	12 156.	8443.94	{8324., 8552.}	30.5369	0.158271	newly 5 weeks spec	2008.	903.86	{858., 949.}	54.9871	0.347001	newly 5 weeks cumul	16 586.	10 660.1	{10 546., 10 778.}	35.7283	0.192002	newly 47 weeks cumul	22 880.	12 599.5	{12 401., 12 774.}	44.9323	0.259133
14	newly 3 weeks spec	249.	166.17	{130., 197.}	33.3614	0.181184	newly 3 weeks cumul	1934.	1512.8	{1358., 1650.}	21.7787	0.107927	newly 5 weeks spec	37.	29.1	{15., 43.}	30.5946	0.168277	newly 5 weeks cumul	2082.	1611.41	{1455., 1762.}	22.6028	0.112669	newly 52 weeks cumul	2131.	1630.95	{1473., 1790.}	23.4655	0.117555
15	newly 3 weeks spec	658.	463.5	{427., 501.}	29.5593	0.153011	newly 3 weeks cumul	3705.	2822.3	{2698., 2920.}	23.8246	0.11839	newly 5 weeks spec	197.	137.38	{113., 159.}	30.3553	0.160275	newly 5 weeks cumul	4246.	3213.3	{3060., 3328.}	24.3217	0.121244	newly 52 weeks cumul	4525.	3379.55	{3228., 3506.}	25.3138	0.126994
16	newly 3 weeks spec	902.	1070.98	{1029., 1120.}	18.7339	0.074362	newly 3 weeks cumul	5020.	5777.86	{5711., 5841.}	15.0968	0.061045	newly 5 weeks spec	342.	382.1	{352., 413.}	11.8187	0.0478347	newly 5 weeks cumul	5877.	6805.19	{6727., 6882.}	15.7936	0.0636655	newly 52 weeks cumul	6347.	7375.26	{7284., 7460.}	16.2007	0.0651902
17	newly 3 weeks spec	2633.	1963.87	{1915., 2011.}	25.4132	0.127431	newly 3 weeks cumul	10 424.	8578.28	{8485., 8659.}	17.7064	0.0846516	newly 5 weeks spec	1461.	961.11	{918., 1010.}	34.2156	0.182162	newly 5 weeks cumul	13 884.	10 915.8	{10 784., 11 026.}	21.3785	0.104476	newly 47 weeks cumul	18 108.	13 091.8	{12 893., 13 251.}	27.7013	0.140892
18	newly 3 weeks spec	1965.	1585.64	{1524., 1639.}	19.3059	0.0933638	newly 3 weeks cumul	8760.	7394.57	{7278., 7513.}	15.5871	0.073625	newly 5 weeks spec	847.	692.03	{654., 738.}	18.2963	0.0882815	newly 5 weeks cumul	10 903.	9137.76	{8979., 9295.}	16.1904	0.0767406	newly 48 weeks cumul	12 657.	10 449.6	{10 239., 10 642.}	17.4405	0.0832807
19	newly 3 weeks spec	2260.	1747.06	{1684., 1805.}	22.6965	0.111935	newly 3 weeks cumul	9867.	8302.09	{8210., 8387.}	15.86	0.0750116	newly 5 weeks spec	1026.	749.81	{709., 785.}	26.9191	0.136536	newly 5 weeks cumul	12 362.	10 196.2	{10 109., 10 287.}	17.5199	0.0836612	newly 50 weeks cumul	14 680.	11 599.3	{11 474., 11 707.}	20.9859	0.102307
20	newly 3 weeks spec	351.	473.82	{450., 499.}	34.9915	0.129894	newly 3 weeks cumul	2657.	3387.09	{3325., 3438.}	27.478	0.105395	newly 5 weeks spec	80.	114.56	{100., 129.}	43.2	0.153693	newly 5 weeks cumul	2903.	3734.96	{3669., 3798.}	28.6586	0.109399	newly 55 weeks cumul	3010.	3843.14	{3774., 3914.}	27.6791	0.106081
21	newly 3 weeks spec	1266.	1072.72	{1022., 1129.}	15.267	0.0722797	newly 3 weeks cumul	6452.	5707.04	{5617., 5801.}	11.5462	0.0533166	newly 5 weeks spec	504.	388.75	{354., 417.}	22.8671	0.113654	newly 5 weeks cumul	7741.	6741.29	{6619., 6872.}	12.9145	0.0600974	newly 52 weeks cumul	8578.	7316.69	{7172., 7500.}	14.704	0.0691272
22	newly 3 weeks spec	1097.	783.93	{738., 828.}	28.5387	0.146409	newly 3 weeks cumul	5662.	4268.75	{4184., 4350.}	24.607	0.122717	newly 5 weeks spec	414.	269.61	{245., 293.}	34.8768	0.187267	newly 5 weeks cumul	6768.	5003.28	{4889., 5102.}	26.0745	0.131257	newly 53 weeks cumul	7400.	5388.8	{5265., 5499.}	27.1784	0.137802
23	newly 3 weeks spec	2274.	1828.44	{1770., 1887.}	19.5937	0.0948356	newly 3 weeks cumul	9195.	7723.32	{7611., 7825.}	16.0052	0.0757741	newly 5 weeks spec	1192.	955.71	{900., 1003.}	19.823	0.096306	newly 5 weeks cumul	12 094.	10 003.9	{9861., 10 119.}	17.2823	0.0824247	newly 46 weeks cumul	15 344.	12 374.7	{12 176., 12 545.}	19.3513	0.0934326
24	newly 3 weeks spec	2818.	2037.67	{1985., 2098.}	27.6909	0.140915	newly 3 weeks cumul	11 319.	8456.2	{8374., 8534.}	25.292	0.126644	newly 5 weeks spec	1477.	1104.3	{1057., 1155.}	25.2336	0.12657	newly 5 weeks cumul	14 831.	11 069.	{10 976., 11 175.}	25.3657	0.127072	newly 46 weeks cumul	19 249.	14 040.2	{13 882., 14 186.}	27.0604	0.137053
25	newly 3 weeks spec	1110.	854.25	{816., 891.}	23.0405	0.114003	newly 3 weeks cumul	5663.	4835.03	{4770., 4903.}	14.6207	0.0686785	newly 5 weeks spec	432.	286.3	{265., 311.}	33.7269	0.179603	newly 5 weeks cumul	6746.	5614.14	{5522., 5702.}	16.7782	0.0797969	newly 49 weeks cumul	7332.	6001.21	{5880., 6111.}	18.1504	0.0870294
26	newly 3 weeks spec	2919.	2575.75	{2518., 2643.}	11.7592	0.0544335	newly 3 weeks cumul	11 387.	10 962.2	{10 874., 11 050.}	3.73039	0.0165206	newly 5 weeks spec	1758.	1321.24	{1276., 1370.}	24.8441	0.124225	newly 5 weeks cumul	15 461.	14 132.8	{14 024., 14 232.}	8.59097	0.0390194	newly 47 weeks cumul	21 236.	17 271.9	{17 113., 17 413.}	18.6671	0.0897454
27	newly 3 weeks spec	2411.	2129.73	{2074., 2180.}	11.6661	0.0539495	newly 3 weeks cumul	9503.	8893.9	{8821., 8969.}	6.40955	0.0287783	newly 5 weeks spec	1418.	1139.94	{1092., 1187.}	19.6093	0.0950438	newly 5 weeks cumul	12 786.	11 602.	{11 511., 11 688.}	9.26044	0.0422112	newly 48 weeks cumul	17 179.	14 627.	{14 506., 14 748.}	14.8556	0.0698539
28	newly 3 weeks spec	2066.	1558.17	{1510., 1609.}	24.5803	0.122691	newly 3 weeks cumul	9289.	7253.23	{7171., 7339.}	21.9159	0.107455	newly 5 weeks spec	1123.	695.14	{660., 739.}	38.0997	0.208762	newly 5 weeks cumul	12 043.	8992.16	{8888., 9107.}	25.3329	0.12689	newly 52 weeks cumul	14 773.	10 360.7	{10 239., 10 492.}	29.8675	0.154098
29	newly 3 weeks spec	2511.	2346.95	{2278., 2410.}	6.53485	0.0294597	newly 3 weeks cumul	10 166.	9747.17	{9645., 9859.}	4.11991	0.0182867	newly 5 weeks spec	1397.	1251.48	{1205., 1298.}	10.4166	0.0479615	newly 5 weeks cumul	13 376.	12 714.	{12 570., 12 837.}	4.94939	0.0220592	newly 46 weeks cumul	17 318.	15 869.4	{15 665., 16 029.}	8.36448	0.0379515
30	newly 3 weeks spec	1594.	1563.49	{1472., 1632.}	2.91782	0.0129696	newly 3 weeks cumul	7832.	7626.72	{7418., 7719.}	2.62104	0.0115789	newly 5 weeks spec	651.	646.58	{585., 691.}	4.57757	0.0202849	newly 5 weeks cumul	9505.	9284.52	{8909., 9405.}	2.31962	0.0102601	newly 55 weeks cumul	10 576.	10 450.2	{9866., 10 618.}	1.39429	0.00620395
31	newly 3 weeks spec	771.	612.44	{570., 651.}	20.5655	0.100514	newly 3 weeks cumul	4574.	3790.82	{3705., 3857.}	17.1224	0.0816183	newly 5 weeks spec	189.	177.99	{160., 201.}	8.54497	0.0391281	newly 5 weeks cumul	5139.	4298.68	{4200., 4388.}	16.3518	0.0776051	newly 56 weeks cumul	5392.	4507.49	{4400., 4597.}	16.4041	0.0778833
32	newly 3 weeks spec	858.	1209.43	{1156., 1257.}	40.9592	0.148821	newly 3 weeks cumul	4649.	6122.79	{6023., 6231.}	31.7012	0.119554	newly 5 weeks spec	288.	467.76	{433., 505.}	62.4167	0.209879	newly 5 weeks cumul	5438.	7345.72	{7222., 7479.}	35.0813	0.13055	newly 53 weeks cumul	5832.	8111.33	{7950., 8273.}	39.0832	0.143212
33	newly 3 weeks spec	3068.	2089.89	{2024., 2146.}	31.881	0.166833	newly 3 weeks cumul	12 287.	9042.42	{8948., 9135.}	26.4066	0.133176	newly 5 weeks spec	1670.	1030.8	{985., 1075.}	38.2754	0.209831	newly 5 weeks cumul	16 122.	11 539.9	{11 425., 11 649.}	28.4213	0.145228	newly 51 weeks cumul	20 713.	13 907.9	{13 776., 14 034.}	32.8544	0.172996
34	newly 3 weeks spec	2614.	2817.84	{2742., 2892.}	7.79801	0.0325218	newly 3 weeks cumul	10 179.	10 115.7	{9987., 10 222.}	0.902446	0.00394094	newly 5 weeks spec	1528.	1915.84	{1858., 1971.}	25.3822	0.0981231	newly 5 weeks cumul	13 712.	14 369.5	{14 181., 14 520.}	4.79514	0.0203263	newly 46 weeks cumul	18 212.	22 040.4	{21 760., 22 338.}	21.0213	0.082841
35	newly 3 weeks spec	2810.	2039.15	{1974., 2098.}	27.4324	0.1394	newly 3 weeks cumul	11 877.	9411.1	{9305., 9506.}	20.762	0.101085	newly 5 weeks spec	1428.	923.17	{879., 962.}	35.3522	0.189716	newly 5 weeks cumul	15 254.	11 706.	{11 571., 11 814.}	23.2594	0.114995	newly 51 weeks cumul	18 586.	13 535.7	{13 350., 13 675.}	27.1725	0.13773
36	newly 3 weeks spec	2377.	2491.16	{1821., 2736.}	14.7623	0.0644855	newly 3 weeks cumul	9678.	10 423.7	{7839., 11 238.}	16.0998	0.0683315	newly 5 weeks spec	1295.	1321.9	{945., 1469.}	13.9537	0.06318	newly 5 weeks cumul	12 751.	13 570.2	{10 071., 14 650.}	15.6036	0.0670954	newly 49 weeks cumul	16 300.	16 903.7	{12 317., 18 307.}	14.4359	0.0639772
37	newly 3 weeks spec	918.	1029.33	{970., 1080.}	13.0795	0.0532999	newly 3 weeks cumul	5030.	5577.15	{5519., 5725.}	11.8459	0.0487113	newly 5 weeks spec	306.	366.49	{331., 401.}	20.1797	0.0788398	newly 5 weeks cumul	5864.	6559.65	{6521., 6711.}	12.7584	0.0522025	newly 54 weeks cumul	6382.	7089.85	{7035., 7255.}	12.1703	0.0500123
38	newly 3 weeks spec	286.	274.91	{241., 303.}	7.18531	0.0324795	newly 3 weeks cumul	2079.	1996.52	{1912., 2082.}	4.29533	0.0192232	newly 5 weeks spec	76.	63.38	{48., 78.}	19.7632	0.100223	newly 5 weeks cumul	2297.	2193.66	{2093., 2299.}	4.74967	0.0213221	newly 54 weeks cumul	2347.	2257.82	{2151., 2373.}	4.29399	0.0192251
39	newly 3 weeks spec	1569.	2163.65	{2092., 2234.}	37.8999	0.139429	newly 3 weeks cumul	6961.	9336.01	{9178., 9499.}	34.1188	0.127455	newly 5 weeks spec	719.	1067.48	{1009., 1118.}	48.4673	0.17132	newly 5 weeks cumul	8738.	11 928.5	{11 748., 12 129.}	36.5128	0.135144	newly 48 weeks cumul	10 021.	14 296.2	{14 105., 14 517.}	42.6623	0.154279
40	newly 3 weeks spec	2069.	2469.71	{2403., 2534.}	19.3673	0.0768024	newly 3 weeks cumul	8421.	10 245.6	{10 157., 10 336.}	21.6671	0.0851628	newly 5 weeks spec	1045.	1337.15	{1277., 1390.}	27.9569	0.106856	newly 5 weeks cumul	10 908.	13 402.7	{13 278., 13 512.}	22.8699	0.089436	newly 49 weeks cumul	13 560.	16 910.2	{16 740., 17 047.}	24.7066	0.0958797
41	newly 3 weeks spec	1243.	1825.86	{1747., 1889.}	46.8914	0.166832	newly 3 weeks cumul	5945.	8444.43	{8329., 8565.}	42.0426	0.152386	newly 5 weeks spec	608.	812.56	{760., 864.}	33.6447	0.125505	newly 5 weeks cumul	7464.	10 486.3	{10 325., 10 642.}	40.4918	0.147619	newly 50 weeks cumul	8541.	12 067.5	{11 855., 12 276.}	41.2893	0.150071
42	newly 3 weeks spec	457.	467.9	{427., 511.}	6.16193	0.026045	newly 3 weeks cumul	2671.	2977.34	{2874., 3092.}	11.4691	0.0469749	newly 5 weeks spec	82.	132.6	{106., 153.}	61.7073	0.20424	newly 5 weeks cumul	2987.	3355.35	{3225., 3487.}	12.3318	0.0503109	newly 54 weeks cumul	3092.	3505.69	{3366., 3655.}	13.3794	0.0543146
43	newly 3 weeks spec	2726.	1303.76	{1247., 1356.}	52.1731	0.320578	newly 3 weeks cumul	11 008.	6102.17	{6003., 6198.}	44.566	0.256257	newly 5 weeks spec	1377.	573.96	{535., 610.}	58.3181	0.380606	newly 5 weeks cumul	14 359.	7542.27	{7427., 7666.}	47.4736	0.279654	newly 47 weeks cumul	18 000.	8651.63	{8490., 8785.}	51.9354	0.318212
44	newly 3 weeks spec	1026.	1129.1	{1088., 1167.}	10.0487	0.0413999	newly 3 weeks cumul	5335.	5657.29	{5599., 5714.}	6.04105	0.0254598	newly 5 weeks spec	393.	452.16	{425., 482.}	15.0534	0.0604393	newly 5 weeks cumul	6350.	6826.95	{6769., 6889.}	7.51102	0.0314433	newly 53 weeks cumul	7041.	7611.78	{7529., 7712.}	8.10652	0.0338346
45	newly 3 weeks spec	374.	421.99	{383., 452.}	12.8957	0.0519073	newly 3 weeks cumul	2546.	2908.35	{2821., 2989.}	14.2321	0.0576751	newly 5 weeks spec	135.	104.37	{89., 121.}	22.7185	0.11528	newly 5 weeks cumul	2913.	3224.02	{3126., 3309.}	10.677	0.0439391	newly 52 weeks cumul	3058.	3331.67	{3218., 3442.}	8.94931	0.0370835
46	newly 3 weeks spec	1322.	1628.25	{1581., 1688.}	23.1657	0.0903294	newly 3 weeks cumul	7028.	8209.81	{8123., 8297.}	16.8157	0.0674838	newly 5 weeks spec	520.	631.57	{594., 672.}	21.4558	0.0838566	newly 5 weeks cumul	8416.	9856.4	{9725., 9973.}	17.115	0.0685918	newly 52 weeks cumul	9218.	10 880.6	{10 721., 11 061.}	18.0363	0.071987
47	newly 3 weeks spec	877.	961.8	{910., 1015.}	9.66933	0.039736	newly 3 weeks cumul	5116.	5273.4	{5161., 5385.}	3.14855	0.0134058	newly 5 weeks spec	280.	331.79	{298., 365.}	18.4964	0.0725095	newly 5 weeks cumul	5861.	6171.39	{6013., 6317.}	5.29756	0.0223401	newly 54 weeks cumul	6385.	6640.71	{6476., 6827.}	4.05967	0.0172062
48	newly 3 weeks spec	1336.	1339.56	{1296., 1380.}	1.98204	0.00857383	newly 3 weeks cumul	7014.	7072.58	{7007., 7144.}	0.97947	0.00422711	newly 5 weeks spec	498.	485.67	{455., 516.}	4.4759	0.0199044	newly 5 weeks cumul	8264.	8367.89	{8291., 8451.}	1.28909	0.00555422	newly 54 weeks cumul	8948.	9103.89	{9011., 9192.}	1.75179	0.00753059
49	newly 3 weeks spec	215.	197.55	{172., 219.}	9.34419	0.0436463	newly 3 weeks cumul	1583.	1575.64	{1521., 1623.}	2.11118	0.00922224	newly 5 weeks spec	43.	42.4	{33., 50.}	14.2791	0.0631056	newly 5 weeks cumul	1735.	1707.66	{1648., 1761.}	2.44035	0.0107713	newly 56 weeks cumul	1768.	1743.56	{1682., 1802.}	2.39819	0.0105652
50	newly 3 weeks spec	2185.	2598.81	{2530., 2666.}	18.9387	0.0752367	newly 3 weeks cumul	9176.	10 815.7	{10 722., 10 893.}	17.8693	0.0713921	newly 5 weeks spec	1236.	1405.01	{1359., 1454.}	13.6739	0.0555044	newly 5 weeks cumul	12 158.	14 137.2	{14 044., 14 236.}	16.2791	0.0654962	newly 47 weeks cumul	15 907.	17 900.2	{17 823., 17 992.}	12.5305	0.0512671
